# Laser-writable high-k dielectric for van der Waals nanoelectronics

**DOI:** 10.1126/sciadv.aau0906

**Published:** 2019-01-18

**Authors:** N. Peimyoo, M. D. Barnes, J. D. Mehew, A. De Sanctis, I. Amit, J. Escolar, K. Anastasiou, A. P. Rooney, S. J. Haigh, S. Russo, M. F. Craciun, F. Withers

**Affiliations:** 1 Centre for Graphene Science, College of Engineering, Mathematics and Physical Sciences, University of Exeter, Exeter EX4 4QF, UK.; 2 School of Materials, University of Manchester, Oxford Road, Manchester M13 9PL, UK.

## Abstract

Similar to silicon-based semiconductor devices, van der Waals heterostructures require integration with high-*k* oxides. Here, we demonstrate a method to embed and pattern a multifunctional few-nanometer-thick high-*k* oxide within various van der Waals devices without degrading the properties of the neighboring two-dimensional materials. This transformation allows for the creation of several fundamental nanoelectronic and optoelectronic devices, including flexible Schottky barrier field-effect transistors, dual-gated graphene transistors, and vertical light-emitting/detecting tunneling transistors. Furthermore, upon dielectric breakdown, electrically conductive filaments are formed. This filamentation process can be used to electrically contact encapsulated conductive materials. Careful control of the filamentation process also allows for reversible switching memories. This nondestructive embedding of a high-*k* oxide within complex van der Waals heterostructures could play an important role in future flexible multifunctional van der Waals devices.

## Teaser

2D material compatible, writable, and high-*k* multifunctional oxide facilitates next-generation flexible van der Waals electronics.

## INTRODUCTION

The high quality of the native oxide that can be grown on the surface of silicon has underpinned the wide success of modern micro- and nanoelectronics. In recent years, high-*k* dielectrics such as HfO_2_ have been adopted to reduce the dimensions of nanoelectronic components and boost their performance (*1*). Recent work has shown similar native oxides in two-dimensional (2D) materials such as HfSe_2_, ZrSe_2_ (*2*), TaS_2_ (*3*) and TaSe_2_ (*4*). However, use of these oxides embedded within van der Waals (vdW) heterostructures has not been shown.

In comparison to silicon, vdW heterostructure devices are likely to play an important role in future electronic device applications (*5*). With a rapidly growing family of layered 2D materials (*6*), the multitude of possible heterostructure combinations available will allow for device designs with unprecedented functionalities and improved performance (*2*). To date, many such vdW heterostructure devices have been shown, such as vertical tunneling transistors (*7*) with negative differential resistance (*8*), light-emitting quantum wells (*9*, *10*), photovoltaics (*11*–*16*) and memory devices (*17*).

Contrary to the conventional molecular beam epitaxy growth of semiconductor devices, vdW heterostructures make it possible to produce atomically sharp interfaces between different materials (i.e., semiconductors, insulators, semimetals, etc.) without concerns for their intercompatibility during fabrication. The absence of dangling bonds on the surface of atomically thin materials allows for the creation of atomically sharp interfaces, eliminating the problem of interdiffusion known to impose severe limitations on the downscaling of devices fabricated by standard semiconductors. To date, the state-of-the-art vdW devices studied experimentally rely on the use of high-purity hexagonal boron nitride (hBN) as a gate dielectric, a tunnel barrier, or a high-quality substrate material (*18*). Such high-quality hBN crystals are not widespread, and scalable chemical vapor deposition versions typically contain impurities that lead to leakage current in transistor devices (*19*, *20*). Furthermore, the dielectric constant of hBN (*k* ≅ 4) is comparable to that of SiO_2_ (*k* = 3.9), thus limiting the downscaling in vdW nanoelectronics (*21*). Common deposition techniques used for SiO_2_ and HfO_2_ are not directly compatible with 2D materials (*22*, *23*). In general, these methods tend to damage or modify the electronic properties of the underlying 2D crystal (*24*), especially when the 2D material is thinned to single-unit cell thickness. Other options include exploring atomically flat layered oxides such as mica or V_2_O_5_ and assembling them layer by layer. However, these dielectrics also result in a significant level of charge transfer to neighboring 2D materials, large hysteresis in field-effect devices, and significant reduction of the mobility (*25*). Therefore, the search for alternative dielectrics or novel technologies, compatible with 2D materials, which give good interface quality and with high -*k*, is needed.

In this work, we demonstrate a route to embed ultrathin HfO_x_ in vdW heterostructures using selective photo-oxidation of HfS_2_. HfS_2_ is a layered semiconductor with an indirect bandgap of 2.85 eV in its bulk form (*26*–*28*) and has comparable surface roughness to other 2D crystals after exfoliation [see the Supplementary Materials for atomic force microscopy (AFM)]. We found that the photo-oxidation process can be enabled using laser light even when the HfS_2_ is embedded within complex heterostructures and under metallic contacts. This fabrication technique eliminates the need for invasive sputtering or atomic layer deposition (ALD) methods (*29*). We demonstrate that the photo-induced HfO_x_ has a dielectric constant *k* ≅ 15 and that this dielectric can be incorporated into four classes of devices enabling different applications: flexible field-effect transistors (FETs), resistive switching random access memories (ReRAMs), vertical light emitters, and photodetectors.

## RESULTS

### Photo-oxidation of HfS_2_ in vdW heterostructures

**Fig. 1:**
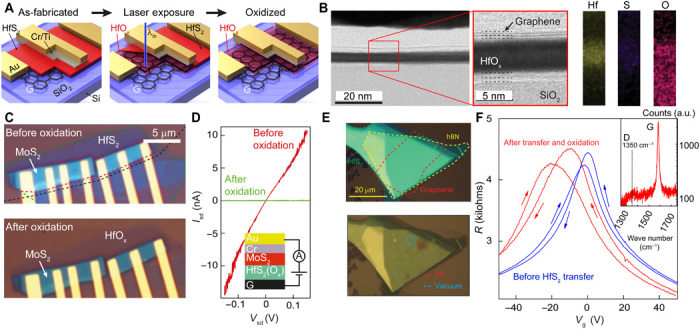
Heterostructure processing and characterization. (**A**) The heterostructure is fabricated via dry transfer peeling from poly(dimethylsiloxane) membrane (left), the area containing HfS_2_ is exposed to laser light (center), and the HfS_2_ is converted into HfO_x_ (right). (**B**) BF STEM image showing a cross section of a Gr/HfO_x_ device after laser-assisted oxidation (left) and EDX elemental analysis (right). a.u., arbitrary units. (**C**) Optical image of a graphene-HfS_2_/MoS_2_ heterostructure before (top) and after (bottom) oxidation. Black outlines the region of the graphene back gate, green outlines the HfO_2_, and red outlines the MoS_2_. (**D**) Current (*I*_sd_) versus applied voltage (*V*_sd_) for the heterostructure in (C) before (red) and after (green) photo-induced oxidation. Inset shows the stacking sequence. (**E**) Top: Optical micrograph of a HfS_2_ flake encapsulated between hBN and graphene (green, HfS_2;_ yellow, hBN; red, graphene). Bottom: Optical micrograph of the same heterostructure imaged within our vacuum chamber showing laser irradiation effects in vacuum (blue hatched area) and in air (red hatched area). Note: No obvious oxidation effects are observed when irradiated in vacuum (*P* ~ 10^−5^ mbar). (**F**) Two-terminal resistance versus gate voltage for a graphene on hBN (*d* ~ 40 nm)/SiO_2_ (290 nm) FET measured at *T* = 266 K in a helium atmosphere (blue curve) and after placing a thin HfS_2_ flake and subjecting it to laser oxidation (red curve; sweep rate = 10 V/min). Inset shows a Raman spectrum of graphene after oxidation plotted on a logarithmic scale showing the G peak and a negligible D peak.

The procedure used to fabricate our devices is illustrated in Fig. 1A. Heterostructures are assembled using dry transfer of micromechanically exfoliated 2D crystals (*25*, *30*, *31*). A few-layer flake of HfS_2_ crystal is placed in the stack where the dielectric is required (Fig. 1A, left). In more complex structures, additional layers are subsequently transferred (see section S1 for details). Once the device has been produced and the contacts have been defined by electron beam lithography, the desired region of oxide is selectively irradiated using visible laser light (Fig. 1A, center and right; see Materials and Methods). It has been shown that, upon laser irradiation, thin HfS_2_ undergoes an oxidation reaction due to the charge transfer between the semiconductor and the water redox couple present on its surface (*32*) and that it converts into an oxide of hafnium.

By comparing laser irradiation effects of HfS_2_ in vacuum and in atmosphere, we show that this process relies on the presence of atmospheric water and/or oxygen (Fig. 1E). Furthermore, the oxidation process is still found to occur even when the HfS_2_ is sandwiched between neighboring 2D materials. Figure 1E shows a region of HfS_2_ stacked within graphene and hBN after laser-assisted oxidation (red hatched region). The mechanism likely involves migration of interfacial water between the graphene-HfS_2_/HfO_x_ interface to the reaction site that is being irradiated. Upon exfoliation, the surface layer of the HfS_2_ will naturally oxidize in the 10 to 15 min before encapsulation, which could allow for diffusion of atmospheric water between the graphene and more hydrophilic HfO_x_ surface. Similar diffusion effects have previously been observed for graphene on SiO_2_ (*33*). Figure 1B shows a high-resolution scanning transmission electron microscopy (HR STEM) image (*34*, *35*) of the cross section of a graphene/HfO_x_/graphene device, where the few-layer top and bottom graphene electrodes are still clearly visible, while the long-range crystal order of HfS_2_ is lost and the resultant material appears in an amorphous phase. Energy-dispersive x-ray spectroscopy (EDX) analysis confirms that the only species present in this phase are hafnium and oxygen, with only low levels of sulfur left after laser irradiation, as shown in Fig. 1B. We find that graphene encapsulated in laser-written HfO_x_ (red curve) shows only a slight reduction in field-effect mobility (~1500 to 5000 cm^2^ V^−1^ s^−1^ at a carrier concentration of ~10^11^ cm^−2^) compared to graphene on our source of hBN (blue curve) and only a small level of n-type doping (Fig. 1F). The inset of Fig. 1F shows the Raman spectrum of graphene encapsulated by HfO_x_ after photo-oxidation, in which a negligible D peak is seen. This indicates that graphene is not significantly structurally damaged by the laser irradiation process.

We characterized the insulating properties of the laser-written HfO_x_ by fabricating a MoS_2_ FET with an 8-nm HfS_2_ flake separating a graphene gate electrode and the Cr/Au contacts, as shown in Fig. 1C. After laser exposure, the transparency of the HfS_2_ film increases significantly, indicating an increase in the bandgap from 2.85 eV, consistent with the formation of an oxide [*E*_g_ ~ 5.5 eV, expected for HfO_x_ (*36*)]. Vertical electron transport through the oxide further supports the transformation to the oxide and indicates that oxidation occurs not only under the flakes of 2D materials but also under the thick Au contacts (*d* = 60 nm) facilitated by diffraction of the laser beam around the approximately micrometer-wide contacts. Figure 1D shows the *I*_sd_-*V*_sd_ characteristics for such a device. Before oxidation, the *I*_sd_-*V*_sd_ shows the typical nonlinear behavior expected for electron transport through a series of semiconducting materials (*37*), with a low-bias vertical resistivity *R* ~ 20 × 10^6^ ohm μm^2^. After oxidation, the resistivity around *V*_sd_ = 0 V increases to *R* ~10^11^ ohm μm^2^, consistent with an increased barrier height.

**Fig. 2:**
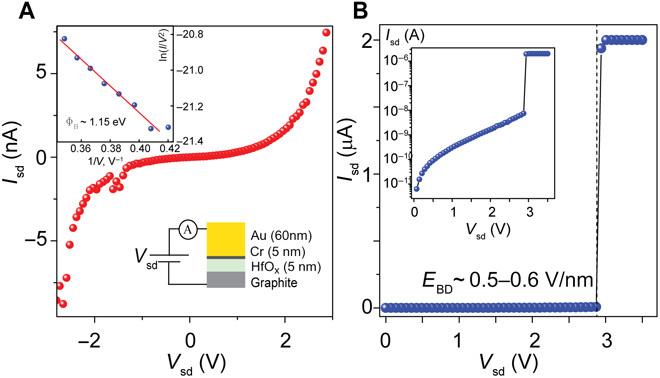
Breakdown of HfO_x_ dielectrics. (**A**) *I*_*s*d_-*V*_sd_ characteristics for a 5-nm graphite-HfO_x_-Cr/Au junction. Top left inset: Fowler-Nordheim tunneling theory. Bottom right inset: Device schematic. (**B**) *I*_sd_-*V*_sd_ for an extended voltage range showing the breakdown field for the dielectric. Inset: Log scale plot of the same data showing the exponential dependence of tunneling current with bias voltage.

The breakdown voltage of the laser-written oxide was measured using a graphite/HfO_x_/Cr (5 nm)/Au (60 nm) vertical electron tunneling device, schematically shown in the inset of Fig. 2A. Tunneling current can be measured when a source-drain bias is applied across the vertical junction, as shown in Fig. 2A. The tunnel current is found to increase exponentially until an electric field of *E_BD_* ~ 0.5 to 0.6 V/nm is applied, at which point the current discontinuously increases to the compliance level of the voltage source meter (Fig. 2B). This breakdown field is comparable to that of SiO_2_ and hBN (0.6 to 2.5 V/nm and 1 V/nm, respectively) (*38*, *39*). The tunnel current is well fit by the Fowler-Nordheim tunneling model (Fig. 2A, inset). We are able to estimate the barrier height value of Φ*_B_* ~ 1.15 eV (*40*). This value is smaller than expected for a graphene-HfO_2_ barrier Φ*_B_* ~ 1.78 eV, likely related to the nonstoichiometry of the amorphous oxide and finite impurity content, leading to an impurity band forming below the conduction band edge. Scaling of the tunnel conductivity with oxide thickness was found to be unreliable with thin oxides *d* < 3 nm displaying significantly lower than expected resistivity (~10^6^ to 10^7^ ohm μm^2^), while oxides of thickness *d* > 10 nm show similar resistivity to 5-nm-thick oxides (~10^11^ to 10^12^ ohm μm^2^). This can be explained as follows: In thin flakes, there is a higher chance for electrical pinholes caused by impurities or defects that shunt the current away from high-resistance paths, whereas thicker flakes do not fully oxidize for the same irradiation energy (which was kept constant in this work), leading to higher than expected conductivity. Optimal thickness for uniform oxidation was found to be 4 to 8 nm. We expect that the oxide quality could easily be improved by optimizing for laser excitation energy, excitation power, and laser spot dwell time during the writing procedure. Current tunneling through thin HfO_x_ dielectric was also measured using conductive AFM (CAFM; see fig. S5).

**Fig. 3:**
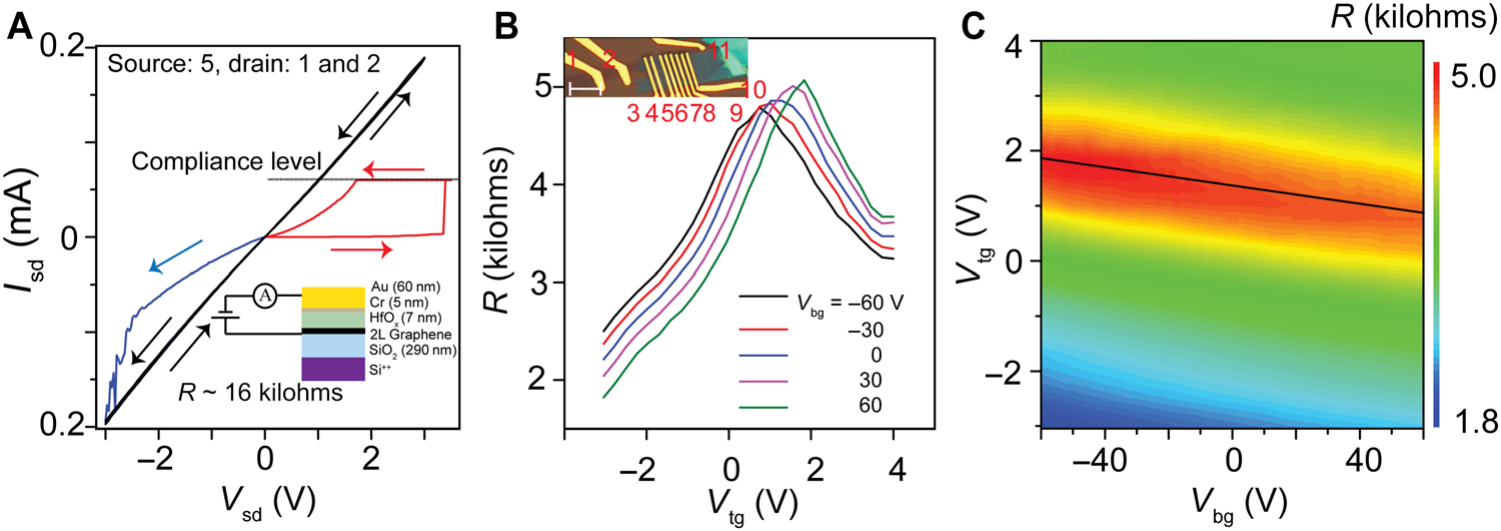
HfO_x_ as an electrical contact material and gate oxide in a dual-gated graphene FET. (**A**) *I*_sd_-*V*_sd_ indicating that the formation of a conductive filament in the oxide (red curve) further reduces resistance to the 10-kilohm level (blue and black curves). (**B**) *R*(*V*_tg_) for different values of *V*_bg_ from −60 to +60 V. Inset: Optical micrograph of the heterostructure device consisting of Gr-HfO_x_-Cr/Au. (**C**) Contour map of the channel resistance between contacts 8 and 10, with contact 9 acting as the top gate electrode.

To better understand the dielectric properties of the laser-written HfO_x_, we fabricated dual-gated graphene FETs. An optical micrograph of a FET constructed on a Si/SiO_2_ (285 nm) substrate from a stack of bilayer graphene/HfO_x_ (7 nm) and Cr/Au contacts is shown in the inset of Fig. 3B. The metal contacts are placed directly on the bilayer graphene (contacts 1, 2, and 11) and on top of the HfO_x_ (contacts 3 to 10). To form a contact between the top Cr/Au metal lead and the graphene underneath the HfO_x_, we rely on the formation of a stable conductive filament produced by the intentional breakdown of the dielectric. In this way, we can use, for example, contacts 7 and 8 in the inset of Fig. 3B as source and voltage probes and contacts 9, 10, and 11 as drain and voltage probes, while the other metal leads (3 to 6 and 8) are used as top gates. The *I*_sd_-*V*_sd_ characteristics showing stable filament formation are shown in Fig. 3A, where the red curve shows the initial dielectric breakdown at a vertical electric field of ~0.5 V/nm. Further cycling of the source-drain bias with increasing current compliance leads to stable nonreversible filament formation that allows for direct contact of the underlying graphene channel. Typical contact resistances of ~5.5 kilohms are achieved after filamentation (as the area of conductive filament is unknown, we cannot estimate the resistivity in this case). Back gate (SiO_2_) sweeps of the resistance show the bilayer graphene to be heavily p-type–doped with the charge neutrality point (CNP) lying at *V*_CNP_ ~ 80 V. These p-type doping levels are attributed to the oxygen plasma cleaning of the Si-SiO_2_ substrate, used to promote the adhesion of graphene before exfoliation. Similar hBN-graphene-HfO_x_ stacks show negligible doping compared to graphene on hBN (Fig. 1F and fig. S6).

Figure 3C shows a four-terminal top gate–back gate contour plot of the four-point channel resistance between contacts 7 and 9 (contacted through filamentation), with contact 8 serving as the top gate electrode. From the slope of the neutrality point, *dV*_tg_/*dV*_bg_, and the thickness of the oxide (determined from the AFM data), we extract the dielectric constant of the HfO_x_ material to be *k* ~ 15 ± 1. This value is similar to literature values for amorphous HfO_x_ (*41*, *42*). Therefore, having confirmed that the dielectric properties of our laser-written HfO_x_ are comparable to those of sputtered HfO_x_ films, we turn our attention to its implementation in electronic devices.

### Laser-written HfO_x_ as high-*k* dielectric for 2D FETs

**Fig. 4:**
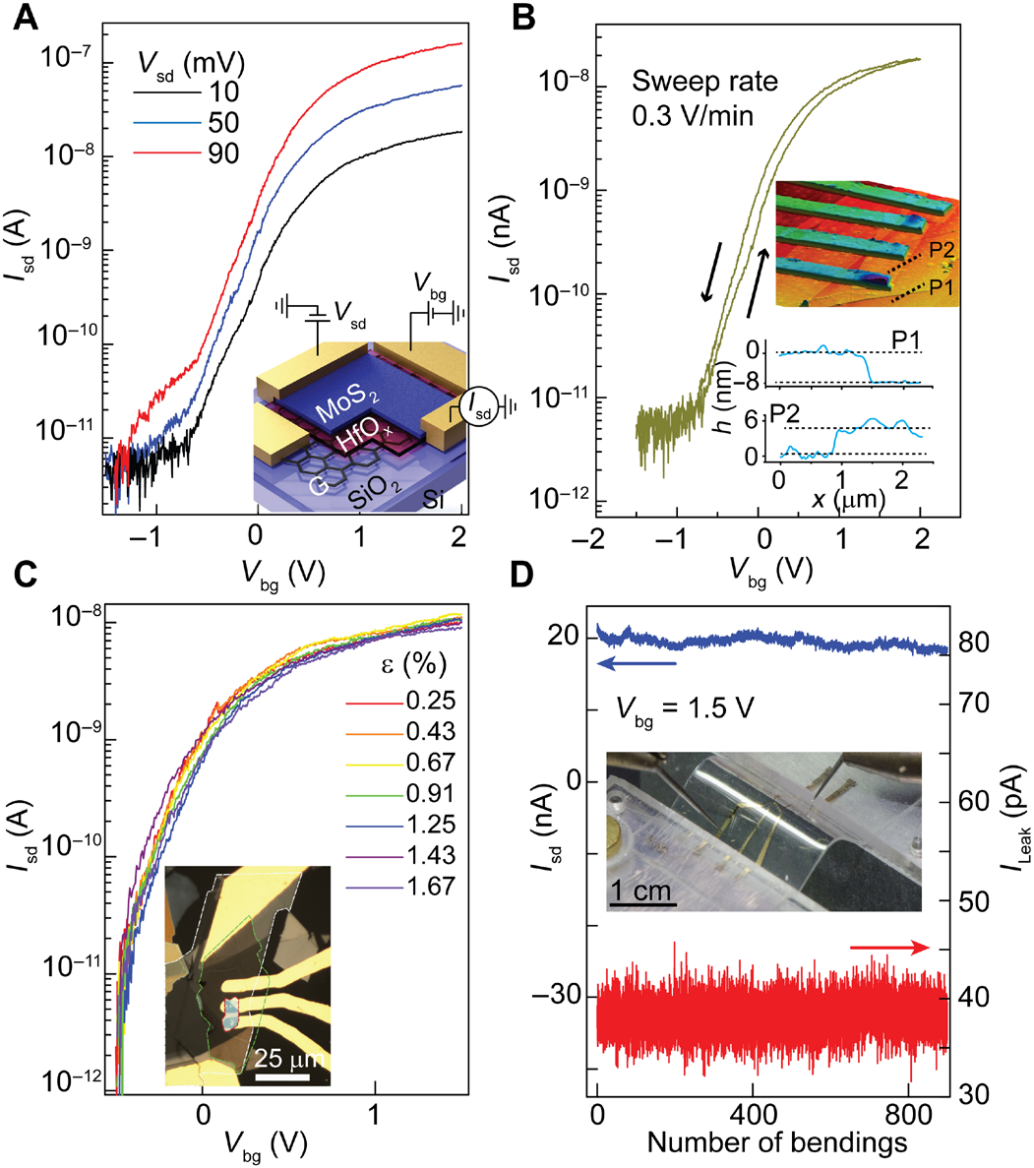
TMDC FETs using photo-oxidized HfS_2_. (**A**) *I*_sd_-*V*_g_ for a MoS_2_ FET with an 8-nm oxidized HfS_2_ film schematically shown in the inset. (**B**) Forward and reverse sweeps highlighting small hysteresis. Inset: The AFM image for the device along with the height profile for the HfO_x_ (P1) and the MoS_2_ (P2). (**C**) Gate voltage dependence of the channel current for a MoS_2_ FET on a 0.5-mm PET substrate for different levels of strain up to 1.6% at *V*_sd_ = 10 mV. Inset: Optical micrograph of the device (white highlight, graphite; green highlight, HfO_x_; and red highlight, MoS_2_). (**D**) Source-drain current at *V*_sd_ = 20 mV (blue) and gate leakage current (red) at *V*_gs_ = 1.5 V over 800 bending cycles.

A drawback of graphene FETs is the absence of a bandgap, which prevents the use of this single layer of carbon atoms in practical FETs, where a suitable *I*_on_/*I*_off_ ratio is required. In contrast, few-layer transition metal dichalcogenides (TMDCs) are semiconductors, and because they are atomically thin (*43*) they are ideally suited for transistor applications. We explored the fabrication of TMDC-FETs using the laser-defined ultrathin high-*k* HfO_x_ on Si/SiO_2_ and flexible polyethylene terephthalate (PET) substrates. We first studied the performance of these devices on a rigid Si/SiO_2_ substrate, as schematically illustrated in the inset of Fig. 4A. Applying a voltage to the graphene electrode (*V*_bg_) allows us to modulate the carrier injection into the MoS_2_ channel. The two-terminal gate dependence of the source-drain channel current (*I*_sd_) for a few-layer MoS_2_ FET at different source-drain bias voltages (*V*_sd_) is shown in Fig. 4 (A and B). We found that these devices have turn-on voltages *V*_g _~ −0.4 V with *I*_on_/*I*_off_ ~10^4^ and subthreshold swings as low as 100 mV per decade. Negligible levels of hysteresis were observed in our device, as shown in Fig. 4B for a sweep rate of 0.3 V/min and *V*_b_ = 10 mV. Higher levels of hysteresis are typically seen for TMDC FETs on SiO_2_ substrates because of the presence of water and oxygen that act as electric field–dependent dopants (*44*–*46*). Field-effect mobilities in the linear region for our MoS_2_ FETs were found to be μ ~ 1 to 2 cm^2^ V^−1^ s^−1^ , comparable to MoS_2_ FETs on SiO_2_ (*47*, *48*). The absence of significant hysteresis highlights the high-quality and low-impurity content of our dielectric. To further understand the level of charge traps in our HfO_x_, we systematically investigated the hysteretic behavior of graphene and MoS_2_ devices in different dielectric environments (see figs. S6 and S7).

To test the suitability of our HfO_x_ for flexible applications, we prepared a multilayer MoS_2_ FET on a 0.5-mm-thick PET substrate and subjected it to uniaxial strains of up to 1.6% in a custom-made bending rig (Fig. 4D, inset). The *I*_sd_-*V*_g_ sweeps are shown in Fig. 4C, where no significant change in the device performance is observed after applying increasing levels of strain. These devices operate over many bending cycles without degradation as shown in Fig. 4D, with a gate leakage current at *V*_g_ = 1.5 V less than 40 pA and a small variation in the *I*_sd_ at a bias voltage of *V*_sd_ = 20 mV.

### ReRAM devices

The formation of conducting filaments illustrated in Fig. 2 allows for switching between two resistance states, creating a device known as ReRAM element (*49*). ReRAM devices represent a promising emerging memory technology with several advantages over conventional technologies including increased speed, endurance, and device density. Of several groups of materials that show resistive switching, transition metal oxides including HfO_x_ are promising candidates (*50*). More recently, these devices based on 2D materials are beginning to attract attention owing to their high mechanical flexibility, reduced power consumption, and potential for high-density memory devices based on stacks of vdW heterostructures (*51*).

**Fig. 5:**
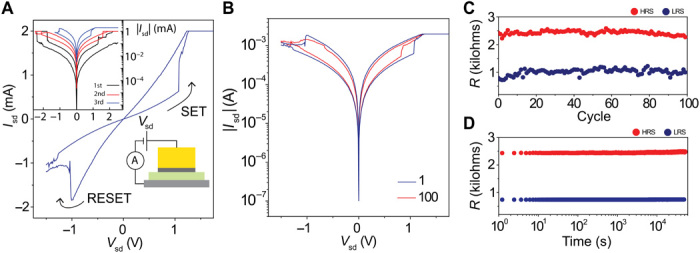
Example of a ReRAM element. (**A**) Example of a switching cycle for the device architecture shown in the bottom right inset. Top left inset: Initial filament formation sweeps before repeatable switching was achieved. (**B**) First and 100th switching cycle for the same device. (**C**) Resistance versus cycle number for the two resistance states plotted for the LRS (blue) and HRS (red). (**D**) Time stability for the two resistance states.

Figure 5 shows representative device characteristics for a typical resistive switching element based on photo-oxidized few-layer HfS_2_. Our devices consist of a Au top electrode with either titanium or chromium used as an adhesion layer deposited on top of the HfS_2_-graphite heterostructure (see Fig. 5A, inset). Following photo-oxidation of the HfS_2_, the device is subjected to repeated current-voltage sweeps, where the top metal electrode is voltage-biased with respect to the bottom graphite electrode. During the initial voltage sweeps, the current compliance and bias voltage are incrementally increased until stable and repeatable resistance cycling is achieved (increasing the current compliance and *V*_b_ further will lead to nonreversible conductive filaments). Figure 5A shows a subsequent switching loop after the initial breakdown. At +1 V, an abrupt increase in current is observed as the device switches from a high-resistance state (HRS) to a low-resistance state (LRS), known as the SET process. The device maintains its LRS as the polarity is reversed and swept down to −1 V, at which a reduction in current for increasing negative voltage is observed, as the device switches back to the HRS, known as the RESET process. The use of thin flakes allows for low-voltage operation, with the SET/RESET voltages around |*V*_sd_| ~ 1 V. The memory window of devices measured here (*R*_HRS_/*R*_LRS_) varies from ~5 up to 10^4^, with the larger values observed for Au/Ti top electrodes (see fig. S8). Figure 5B shows similar current-voltage behavior for the 1st and 100th cycle. The results of repeated cycling are shown in Fig. 5C in which *R*_HRS_/*R*_LRS_ (with both resistance values extracted at *V*_sd_ = 100 mV) shows little variation over 100 cycles. Last, we investigated the long-term stability of this ReRAM device (Fig. 5D) and found that the resistance levels, measured for *V*_sd_ = 250 mV, were consistent and well defined for more than 10^4^ s. We note that resistive switching in devices using graphene for both top and bottom electrodes was unreliable, and we postulate that electrode material asymmetry is crucial for reliable device performance. This bipolar switching is consistent with the formation and rupture of conducting filaments; however, further studies are required to optimize device performance and to better understand the role played by disorder, oxide thickness, and contact chemistry.

### Optoelectronic devices

As discussed above, vertical electron transport in HfO_x_ formed from thin one- to three-layer parent HfS_2_ crystals allowed for much higher tunneling currents. While these high leakage currents are detrimental in transistor applications, other device types require higher electron transparency and higher injection rates. As such, we made use of this property of thinner oxides to realize light-emitting and light-detecting tunneling transistors.

Such vertically stacked heterostructures of 2D materials provide a framework for the creation of large-area, yet atomically thin and flexible optoelectronic devices with photodetectors (*11*, *13*, *52*) and light-emitting diodes (*9*, *10*, *53*). To date, only hBN tunnel barriers have been demonstrated; however, other wide-gap material oxides have not been explored when combined with vdW heterostructures. Here, we demonstrate the use of ultrathin HfO_x_ tunnel barriers in vertical light-emitting tunneling transistor device geometries.

**Fig. 6:**
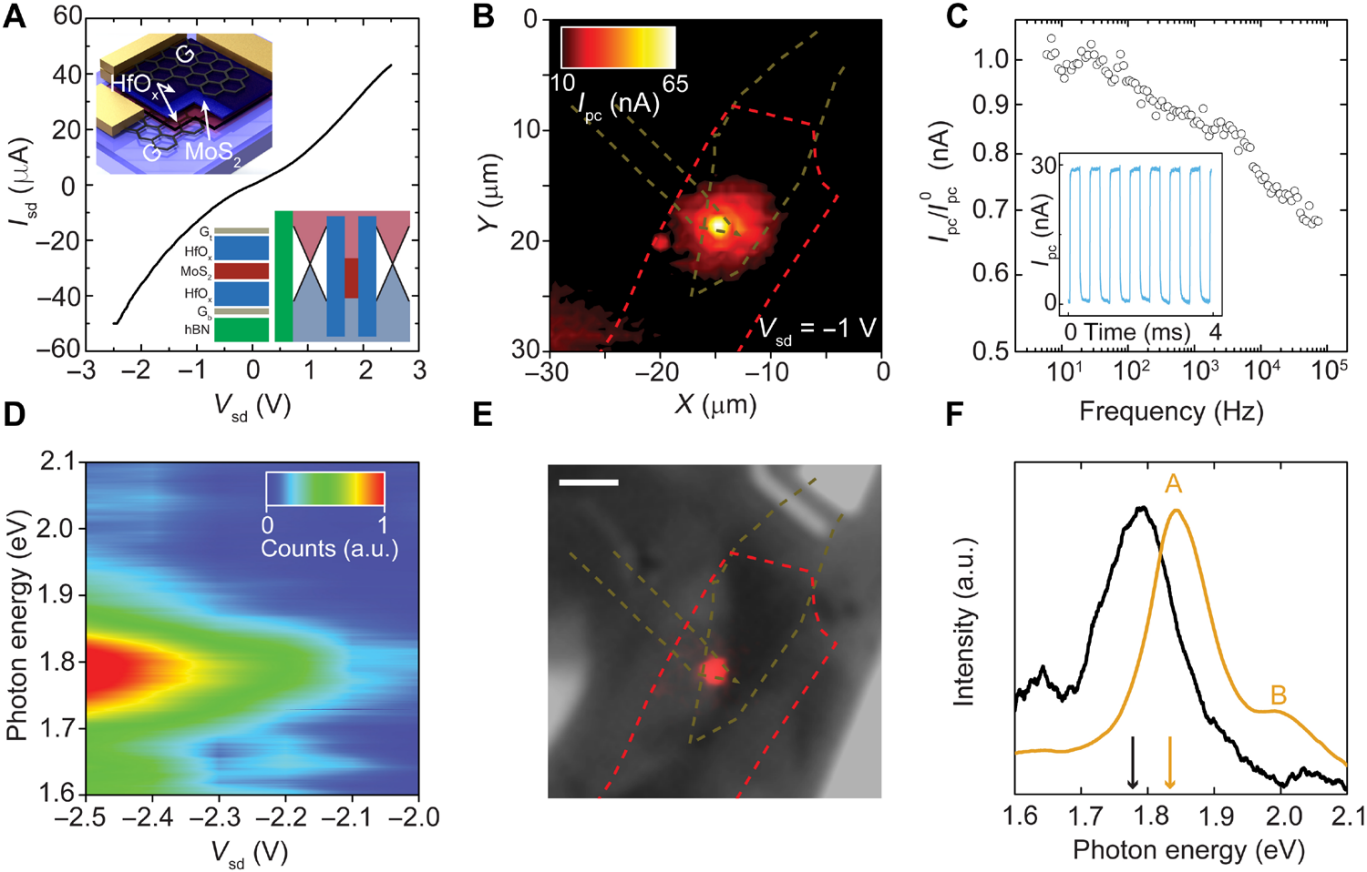
Thin HfO_x_ barriers for optoelectronic applications. (**A**) Current-voltage characteristics for the SQW. Insets: Illustration of the device architecture (top) and schematic of the heterostructure band alignment (hBN-Gr_b_-HfO_x_-MoS_2_-HfO_x_-Gr_t_) (bottom). (**B**) Scanning photocurrent map acquired with a bias of *V*_sd_ = −1 V applied between the top and bottom graphene. (**C**) Normalized photocurrent as a function of modulation frequency. Inset: The temporal response of the photocurrent at *f* = 1.8 kHz. (**D**) Color map of the EL spectra as a function of *V*_sd_. (**E**) False-color charge-coupled device image of the EL overlaid on an optical image of the device. Scale bar, 5 μm. (**F**) Comparison between the normalized intensities of the EL (black) and PL (brown) acquired at *V*_sd_ = −2.5 V and *V*_sd_ = 0 V, respectively.

Figure 6A shows a current-voltage curve of a HfO_x_ single-quantum well (SQW) device formed by the encapsulation of monolayer MoS_2_ in 1 to 2 nm of HfO_x_. Applying a bias voltage between the top and bottom graphene electrodes (*G*_t_ and *G*_b_) allows a current to tunnel through the thin HfO_x_ layers and into the MoS_2_. There is a negligible temperature dependence of the measured source-drain current, indicating a tunneling mechanism rather than transport through low-energy impurity states (see fig. S9). As we increase the bias voltage from zero, the current increases nonlinearly. Outside of a low-bias regime (|*V*_sd_| > 1 V), we observe an increase in the current due to tunneling into the conduction band of MoS_2_. In addition, an asymmetry between the current at positive and negative bias voltage is observed, which is likely due to both a variation in doping between *G*_t_ and *G*_b_ and a different thickness of the top and bottom HfO_x_. This behavior is similar to previous work using hBN tunnel barriers (*9*).

To determine the active area of the heterostructure, we used scanning photocurrent microscopy, whereby a laser beam is rastered across the device while photocurrent is acquired simultaneously (see Materials and Methods). Figure 6B shows that, under a moderate bias (*V*_sd_ = −1 V), the photocurrent is predominately localized to regions of overlap between the top and bottom graphene flakes, each outlined in light green. Photoexcited carriers in MoS_2_ (red outline) are separated by the graphene electrodes because of the applied vertical electric field. Away from this region, the photocurrent (*I*_pc_) drops from >65 to <10 nA. In Fig. 6C, we measure a reduction in the magnitude of the photocurrent as we increase the light modulation frequency. By normalizing this to the value of the photocurrent at low frequencies *I*_pc_^0^, we can ascertain the −3-dB bandwidth of the device, which we found to be *f*_−3dB_ = 40 kHz. From this, we can estimate the rise time using *t*_r_ = 0.35/*f*_−3dB_ ~ 8.8 μs, which is in agreement with our analysis of the temporal response of the photocurrent (see the Supplementary Materials). The inset of Fig. 6C shows multiple iterations of the photocurrent obtained at 1.8 kHz. The measured response time is 10^3^ to 10^6^ times faster than that of typical planar MoS_2_ photodetectors (*54*), a result arising from the use of a vertical, as opposed to lateral, contact geometry. The small electrode separation of ~6 nm and large electric fields of ~0.1 to 0.2 V/nm minimize the transit time of the photoexcited carriers. Hence, these vertical heterostructures of MoS_2_ encapsulated in HfO_x_ are a promising high-speed light-detection architecture.

As the bias voltage is further increased, the quasi–Fermi levels of the graphene electrodes allow for simultaneous injection of electrons into the conduction band of MoS_2_ and holes into the valence band. The carrier confinement set by the HfO_x_ tunnel barriers allows for exciton formation in the MoS_2_. The subsequent decay of those excitons leads to light emission at the excitonic gap of MoS_2_. Figure 6D shows the electroluminescence (EL) intensity map as a function of photon energy and bias voltage, where the main EL band appears at 1.78 eV. Line plots of the EL spectra at 0.1-V increments are shown in the Supplementary Materials.

Briefly, EL is not observed when *V*_sd_ > −2 V. Only upon reducing the bias voltage below −2 V can EL be detected with a more intense signal recorded by increasing |*V*_sd_|. The emergence of EL at −2 V corresponds well with the single-particle bandgap of monolayer MoS_2_ (*55*, *56*) while the negative threshold voltage can be attributed to the asymmetric device structure.

Figure 6E shows a false-color charge-coupled device image of the EL overlaid on a monochrome image of the device at an applied bias voltage of −2.5 V. The EL is localized to the active area of the device previously identified in Fig. 6B through photocurrent mapping. To further understand the emission, normalized EL and photoluminescence (PL) spectra are shown in Fig. 6F. The main PL emission peak is assigned to the A exciton seen at an energy of 1.8 eV. The energy of the main EL band red-shifts from that of PL by 53 meV. Typically, the exfoliated monolayer MoS_2_ is n-doped, which favors the formation of negatively charged excitons (*57*), which have a lower emission energy than that of the neutral exciton by ~30 meV. Therefore, we attribute the main feature in EL spectra at 1.78 eV to the radiative recombination of the charged exciton. Moreover, the dissociation energy (i.e., energy shift referring to that of neutral exciton) of the charged exciton is proportional to the doping concentration (*57*). Therefore, it is likely that the large energy difference between EL and PL is an indication of high doping in monolayer MoS_2_, which is due to doping of the as-exfoliated natural MoS_2_ flakes.

## CONCLUSION

In conclusion, we show that ultrathin few-layer HfS_2_ can be incorporated into a variety of vdW heterostructures and selectively transformed into an amorphous high-*k* oxide using laser irradiation. In contrast to sputtering or ALD, the use of photo-oxidized HfS_2_ allows for clean interfaces without damaging the underlying 2D materials. We demonstrate that the laser-written HfO_x_ has a dielectric constant *k* ~ 15 and a breakdown field of ~0.5 to 0.6 V/nm. These properties allow us to demonstrate several promising high-quality vdW heterostructure devices using this oxide: (i) ReRAM elements that operate in the voltage limit of ~1 V; (ii) flexible TMDC-FETs with *I*_on_/*I*_off_ > 10^4^, subthreshold swings of 100 mV per decade, and good resilience to bending cycles; and (iii) optoelectronic devices based on quantum well architectures, which can emit and detect light in the same device, with EL intensities and drive voltages comparable to devices with hBN barriers and photodetection response times up to 10^6^ times faster than equivalent planar MoS_2_ devices. Moreover, the high-*k* dielectric constant, the compatibility with 2D materials, and the ease of laser-writing techniques (*58*) will allow for significant scaling improvements and greater device functionality, which we predict to be an important feature for future flexible semi-transparent vdW nanoelectronics.

## MATERIALS AND METHODS

### Device fabrication

Devices were fabricated using standard mechanical exfoliation of bulk crystals and dry transfer methods used to form the heterostructures (see the Supplementary Materials for details). Following heterostructure production, the contacts were structured using either optical or electron beam lithography, followed by thermal evaporation of Cr/Au (5/60 nm) electrodes.

After vdW assembly, photo-oxidation of the HfS_2_ layer was performed by rastering either ultraviolet (UV) (λ_in_ = 375 nm) or visible (λ_in_ = 473 nm) laser light focused to a diffraction-limited spot in a custom-built setup (*59*). A typical energy density of 53 mJ/μm^2^ was used for exposures lasting 1 to 2 s per point of the HfS_2_ layer. The focused spot size was *d*_s_ = 264 nm for the UV laser and *d*_s_ = 445 nm for the visible wavelength.

### Materials characterization

#### STEM imaging

A cross-sectional specimen for HR STEM was prepared in an FEI Dual Beam Nova 600i instrument incorporating a focused ion beam and a scanning electron microscope in the same chamber. Using 30-kV ion milling, platinum deposition, and lift-out with a micromanipulator, a thin cross section of material was secured on an Omniprobe TEM grid and thinned down to electron transparency with low-energy ions. HR STEM images were acquired using a probe side aberration-corrected FEI Titan G2 80 to 200 kV with an X-FEG electron source. Bright-field (BF) imaging and high-angle annular dark-field (HAADF) imaging were performed at 200 kV using a probe convergence angle of 21 mrad, an HAADF inner angle of 48 mrad, and a probe current of ~80 pA. The lamellae were aligned with the basal planes parallel to the incident electron probe. Correct identification of each atomic layer within BF and HAADF images was achieved by elemental analysis with EDX spectrum imaging.

#### Atomic force microscopy

AFM was performed using a Bruker Innova system operating in the tapping mode to ensure minimal damage to the sample’s surface. The tips used were Nanosensors PPP-NCHR, which have a radius of curvature smaller than 10 nm and operate in a nominal frequency of 330 kHz.

### Electrical measurements

*I*_sd_-*V*_sd_ values were collected using a Keithley 2400 voltage/current source meter. Electrical characterization of graphene and TMDC FETs was performed using standard low-noise AC lock-in techniques using a Signal Recovery 7225 lock-in amplifier and a Keithley 2400 source meter providing the gate voltage.

All electrical transport measurements were performed in either a vacuum of 10^−3^ mbar or a dry helium atmosphere at room temperature, unless otherwise stated. The flexible MoS_2_ FET produced on PET was measured under ambient conditions.

### Optoelectronic characterization

Optoelectronic measurements were performed using a custom-built setup (*59*). Photocurrent measurements were performed using a continuous-wave laser (λ_in_ = 514 nm, *P* = 15 W/cm^2^) rastered on the devices to produce spatial maps of the photoresponse. The electrical signal was acquired by a DL Instruments Model 1211 current amplifier connected to a Signal Recovery model 7124 digital signal processing lock-in amplifier. The frequency modulation of the lasers was 73.87 Hz. EL and PL measurements were performed in the same setup using a Princeton Instruments SP2500i spectrometer and a PIXIS400 camera. All measurements were performed at room temperature in vacuum (*P* = 10^−5^ mbar).

## Supplementary Material

http://advances.sciencemag.org/cgi/content/full/5/1/eaau0906/DC1

## SUPPLEMENTARY MATERIALS

Supplementary material for this article is available at http://advances.sciencemag.org/cgi/content/full/5/1/eaau0906/DC1 Section S1. Device fabrication Section S2. HR STEM of heterostructure devices Section S3. Atomic force microscopy Section S4. Conductive AFM Section S5. Hysteresis of graphene and MoS_2_ FETs Section S6. Further examples of ReRAM elements with titanium adhesion layer Section S7. Additional optoelectronic device data Fig. S1. Heterostructure processing route. Fig. S2. Additional TEM data. Fig. S3. AFM data. Fig. S4. Comparison of surface roughness of graphene on hBN and on HfS_2_. Fig. S5. CAFM on HfO_x_. Fig. S6. Hysteresis behavior of graphene and MoS_2_ FETs in different dielectric environments. Fig. S7. Comparison of hysteresis width (Δ*V*_H_) as a function of sweep rate for the hBN-MoS_2_-HfO_x_ and SiO_2_-MoS_2_-HfO_x_ devices. Fig. S8. Additional ReRAM devices. Fig. S9. Temperature dependence of the resistance for a graphite-HfO_x_-Cr/Au vertical structure with *t* < 3 nm tunnel barriers. Fig. S10. Additional optoelectronic characterization. References (*60*, *61*)
