# Supplementary material for: Laser-writable high-k dielectric for van der Waals nanoelectronics
Source: Sci Adv. 2019 Jan 18;5(1):eaau0906. doi: 10.1126/sciadv.aau0906 (PMC6357741; doi:10.1126/sciadv.aau0906)
Supplement: http://advances.sciencemag.org/cgi/content/full/5/1/eaau0906/DC1 [file supp_5_1_eaau0906__index.html]

Science Advances | Science Advances

## Supplementary Materials

**This PDF file includes:**

- Section S1. Device fabrication
- Section S2. HR STEM of heterostructure devices
- Section S3. Atomic force microscopy
- Section S4. Conductive AFM
- Section S5. Hysteresis of graphene and MoS2 FETs
- Section S6. Further examples of ReRAM elements with titanium adhesion layer
- Section S7. Additional optoelectronic device data
- Fig. S1. Heterostructure processing route.
- Fig. S2. Additional TEM data.
- Fig. S3. AFM data.
- Fig. S4. Comparison of surface roughness of graphene on hBN and on HfS2.
- Fig. S5. CAFM on HfOx.
- Fig. S6. Hysteresis behavior of graphene and MoS2 FETs in different dielectric environments.
- Fig. S7. Comparison of hysteresis width (Δ*V*H) as a function of sweep rate for the hBN-MoS2-HfOx and SiO2-MoS2-HfOx devices.
- Fig. S8. Additional ReRAM devices.
- Fig. S9. Temperature dependence of the resistance for a graphite-HfOx-Cr/Au vertical structure with *t* < 3 nm tunnel barriers.
- Fig. S10. Additional optoelectronic characterization.
- References (*60*, *61*)

Download PDF

**Files in this Data Supplement:**

- Adobe PDF - aau0906\_SM.pdf
