# Supplementary material for: Laser-writable high-k dielectric for van der Waals nanoelectronics
Source: Sci Adv. 2019 Jan 18;5(1):eaau0906. doi: 10.1126/sciadv.aau0906 (PMC6357741; doi:10.1126/sciadv.aau0906)
Supplement: http://advances.sciencemag.org/cgi/content/full/5/1/eaau0906/DC1 [file aau0906_SM.pdf]

Science Advances
AAAS

advances.sciencemag.org/cgi/content/full/5/1/eaau0906/DC1

Supplementary Materials for

Laser-writable high-*k* dielectric for van der Waals nanoelectronics

N. Peimyoo, M. D. Barnes, J. D. Mehew, A. De Sanctis, I. Amit, J. Escolar, K. Anastasiou, A. P. Rooney, S. J. Haigh, S. Russo, M. F. Craciun, F. Withers*

*Corresponding author. Email: f.withers2@exeter.ac.uk

Published 18 January 2019, *Sci. Adv.* **5**, eaau0906 (2019)
DOI: 10.1126/sciadv.aau0906

This PDF file includes:

Section S1. Device fabrication
Section S2. HR STEM of heterostructure devices
Section S3. Atomic force microscopy
Section S4. Conductive AFM
Section S5. Hysteresis of graphene and MoS_2 FETs
Section S6. Further examples of ReRAM elements with titanium adhesion layer
Section S7. Additional optoelectronic device data
Fig. S1. Heterostructure processing route.
Fig. S2. Additional TEM data.
Fig. S3. AFM data.
Fig. S4. Comparison of surface roughness of graphene on hBN and on HfS_2.
Fig. S5. CAFM on HfO_x.
Fig. S6. Hysteresis behavior of graphene and MoS_2 FETs in different dielectric environments.
Fig. S7. Comparison of hysteresis width (ΔV_H) as a function of sweep rate for the hBN-MoS_2-HfO_x and SiO_2-MoS_2-HfO_x devices.
Fig. S8. Additional ReRAM devices.
Fig. S9. Temperature dependence of the resistance for a graphite-HfO_x-Cr/Au vertical structure with $t < 3$ nm tunnel barriers.
Fig. S10. Additional optoelectronic characterization.
References (*60, 61*)

Section S1. Device fabrication

We make use of a PDMS stamp transfer technique (*30*). Figure S1 shows a typical fabrication route for a MoS_2 FET. Firstly, graphene is mechanically exfoliated onto a thermally oxidized silicon wafer. After this the HfS_2 flakes are exfoliated onto PDMS and transferred to the graphene. The HfS_2 flakes are released from the PDMS between 50-60°C. This process is then repeated for the subsequent layers of the device as shown in fig. S1C. After the heterostructure stack is formed conventional electron beam lithography is used to define electrical contacts.

The same process is used for other devices such as memory devices, dual gated graphene FET's and light emitting quantum well devices.

For devices on hBN substrates we use a PMMA membrane and dry peel the graphene from the PMMA onto the hBN (*25, 31*).

In this work the HfS_2 and WSe_2 was purchased from HQGraphene (http://www.hqgraphene.com/) whilst the MoS_2 and hBN crystals were acquired from Manchester Nanomaterials (http://mos2crystals.com/).

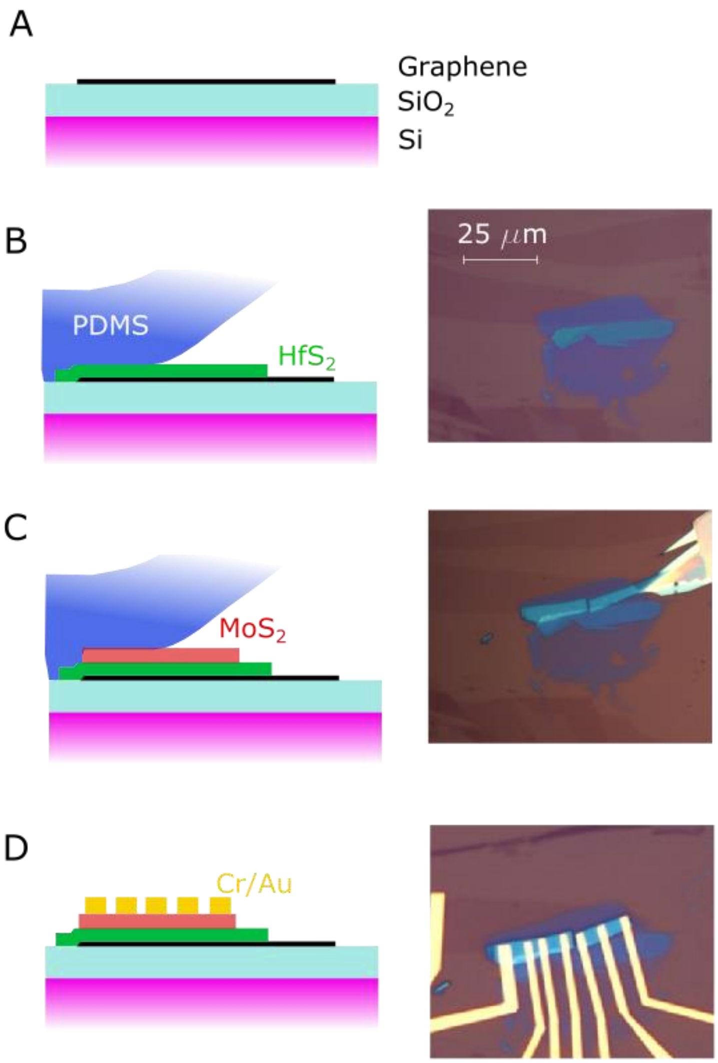

Fig. S1. Heterostructure processing route. (**A**) Graphene/graphite mechanically exfoliated onto a thermally oxidized silicon wafer with SiO_2 thickness of 290 nm (**B**) These HfS_2 flakes are transferred using a PDMS stamp onto the graphene (**C**) Graphene or TMDC's are then transferred by PDMS onto the HfS_2 layer. (**D**) Conventional mico-fabrication of Cr(5nm)/Au(50nm) contacts to the graphene/graphite back-gate and to the TMDC channel. Followed by plasma etching in O_2/Ar plasma.

Section S2. HR STEM of heterostructure devices

Figure S2 shows additional scanning transmission electron microscope (STEM) data for a cross-sectional device based on a Gr-HfO_x-Gr heterostructure The EDX spectroscopy elemental mapping confirms the structure and composition, see fig. S2A. Figure S2B is an optical image of the irradiated device imaged in A. Figure S2(C-E) shows scanning electron microsc (SEM) images illustrating the process of TEM sample preparation using FIB.

The details of cross-sectional STEM sample preparation can be found in previous reports (*9, 34*).

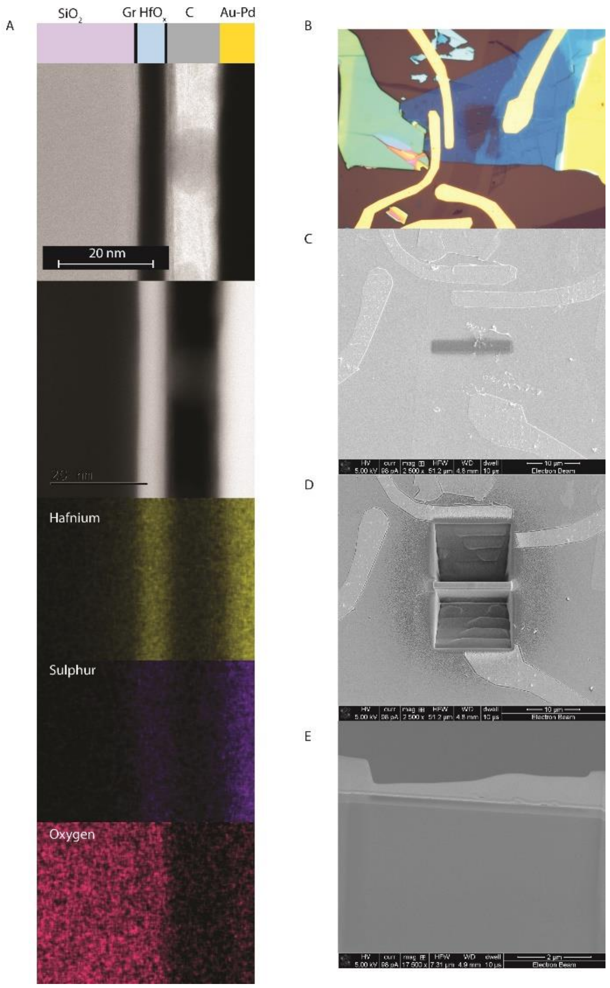

Fig. S2. Additional TEM data. (**A**) Cross-section of a Graphene-HfO_x-Graphene heterostructure along with EDX spectroscopy elemental mapping of the heterostructure showing the HfO_x layer and low sulphur content. (**B**) Optical micrograph of the device imaged in A, the central transparent region has been transformed into amorphous HfO_x via laser irradiation. (**C** to **E**) SEM images of during FIB milling of the TEM sample.

Section S3. Atomic force microscopy

Tapping mode atomic force microscopy (AFM) was used to extract the flake thicknesses in different devices. Figure S3 shows the dual gated graphene FET shown in Fig. 3 of the main text. The average thickness obtained from several cross-sections was 7.4 nm.

AFM can also be used to understand how clean the interface is formed between two materials. fig. S4 shows a hBN-HfS_2-Graphene heterostructure. We find that the roughness of graphene on HfS_2 is comparable to that of graphene on hBN.

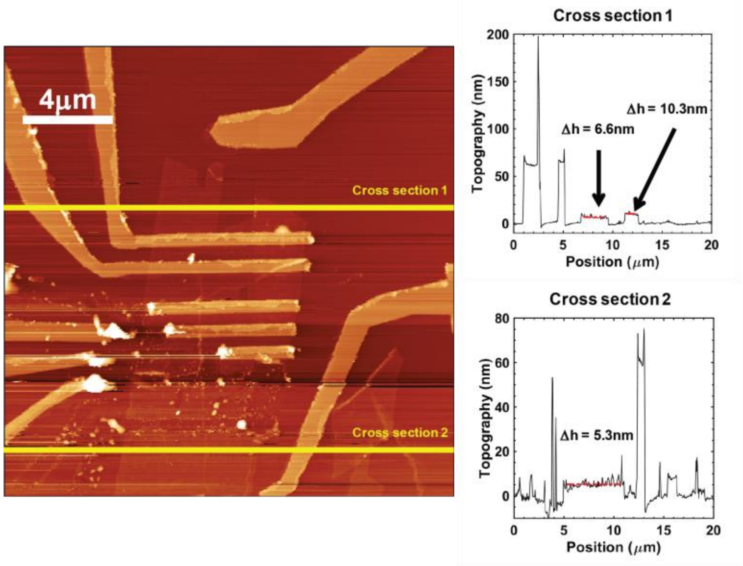

Fig. S3. AFM data. (Left) AFM topography of the dual gated bilayer graphene FET shown in Figure 3 of the main text. (Right) cross-sections of the height profile for the HfO_x dielectric.

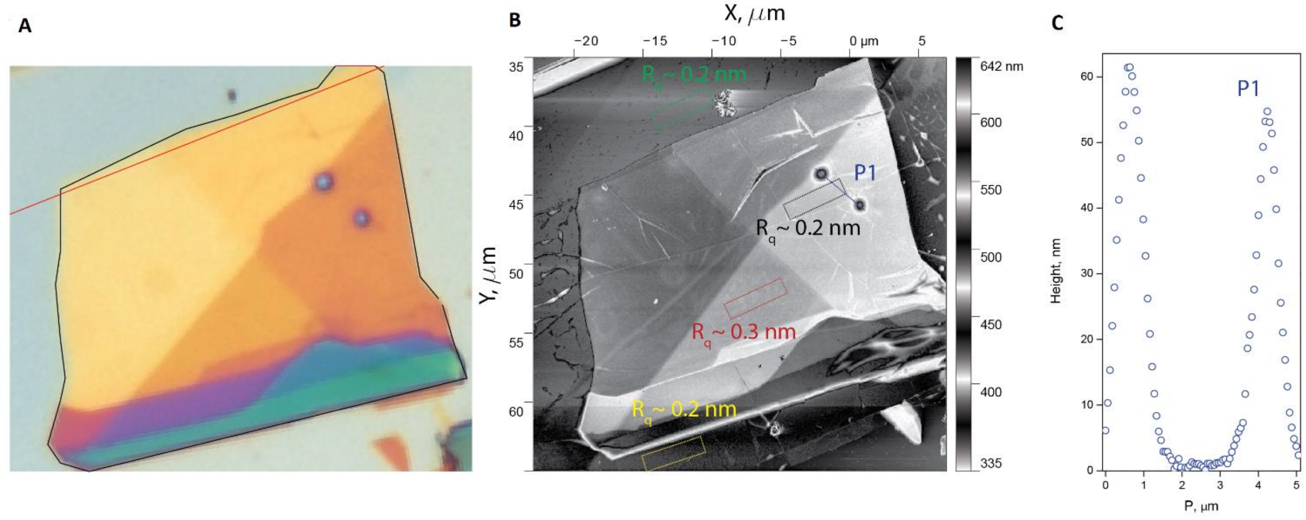

Fig. S4. Comparison of surface roughness of graphene on hBN and on HfS_2. (**A**) Optical image of the hBN-HfS_2-Graphene heterostructure stack. (**B**) Tapping mode atomic force microscopy of a graphene encapsulated hfS_2 flake (note, the RMS roughness, R_q of the hBN (green), graphene on hBN (yellow) and graphene on HfS_2 (black and red) are of the same order ~ (0.2-0.3) nm) and (**C**) height profile of bubbles of contamination trapped between the hBN-HfS_2 interface, marked P1 in (B).

Section S4. Conductive AFM

Conductive AFM was used to locally probe the tunneling current through ultrathin HfO_x. A voltage was applied to the graphite/Au substrate and the current measured using a conductive tip (diamond like carbon) connected to a Femto DLPCA current amplifier and voltmeter. Figure S5 shows multiple I-V curves for a 2.3 nm thick flake of HfO_x. Topographical image analysis and height profile extraction were performed with WSxM v9.1 software (*60*).

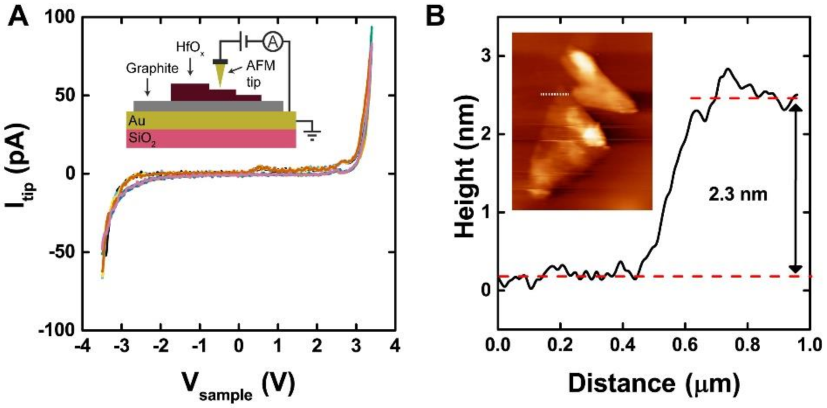

Fig. S5. CAFM on HfO_x. (**A**) Multiple IV curves acquired on flake by applying a voltage to the sample and measuring the current with the AFM tip. Inset shows measurement schematic. (**B**) Height profile of flake. Inset is topographical image of sample

Section S5. Hysteresis of graphene and MoS_2 FETs

To further investigate the quality of our photo-oxidized HfO_x, we fabricate three different heterostructure FETs (i) hBN-graphene-HfO_x (ii) hBN-MoS_2-HfO_x (iii) SiO_2-MoS_2-HfO_x and the hysteresis of these devices are measured with different sweep rates (fig. S6). We find large hysteresis in SiO_2-MoS_2-HfO_x device and the hysteresis width (ΔV_H) increases significantly, whilst ΔV_H is reduced for MoS_2 encapsulated between hBN and HfO_x. An increase in hysteresis of MoS_2 in contact with SiO_2 is consistent with previous reports, which is originated from charge traps at the interface between MoS_2 and SiO_2. We also observe a negligible level of hysteresis for a graphene transistor encapsulated between hBN and HfO_x.

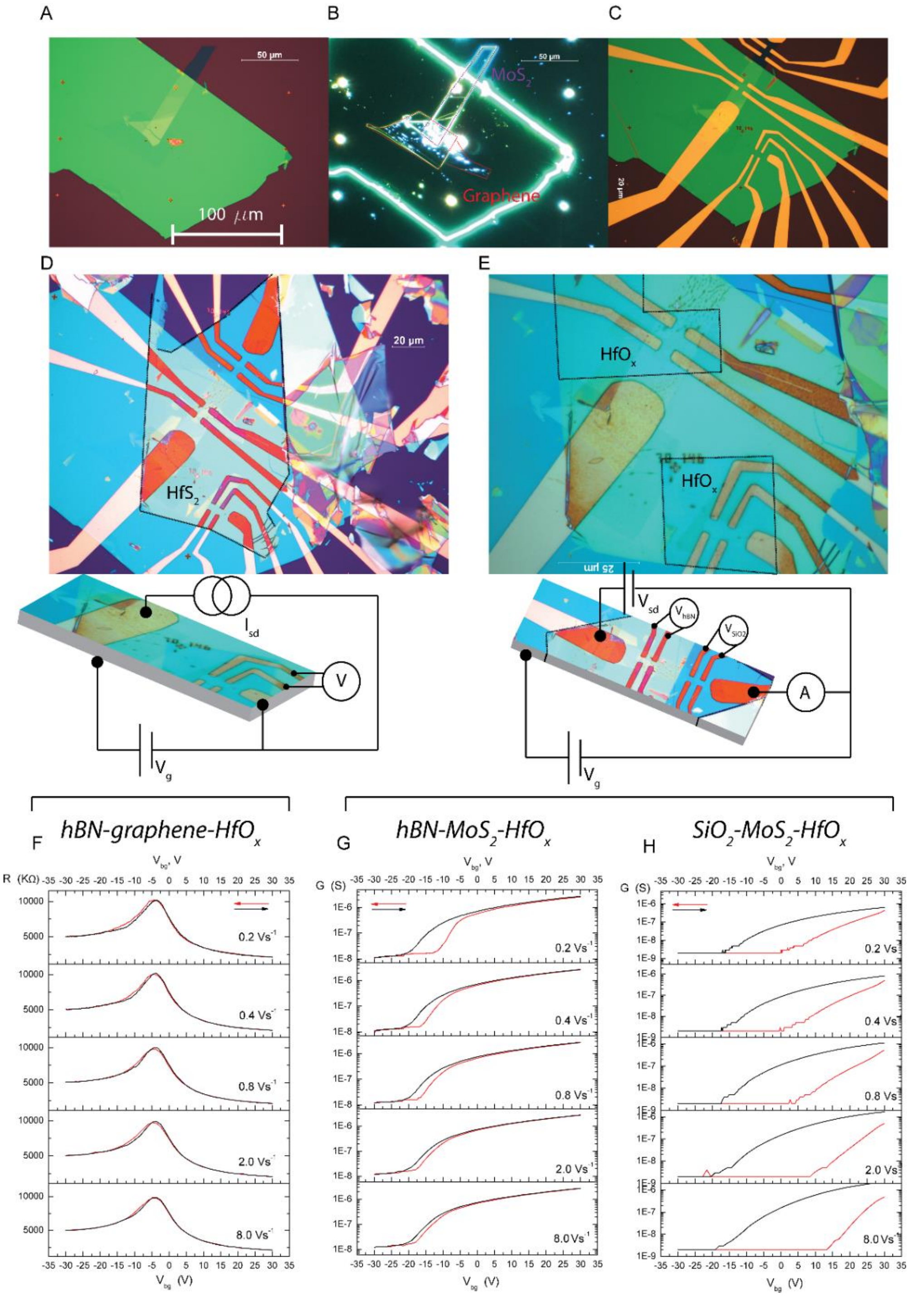
A
B
C
50 µm
100 µm
MoS2
Graphene
20 µm
D
E
HfS2
HfOx
HfOx
25 µm
Isd
V
Vg
Vsd
VhBN
VSiO2
A
Vg
hBN-graphene-HfOx
hBN-MoS2-HfOx
SiO2-MoS2-HfOx
F
G
H
R (KΩ)
G (S)
G (S)
Vbg, V
Vbg (V)
0.2 Vs-1
0.4 Vs-1
0.8 Vs-1
2.0 Vs-1
8.0 Vs-1

Fig. S6. Hysteresis behavior of graphene and MoS_2 FETs in different dielectric environments. (**A**) Optical image of heterostructures consisting of different stacking sequences. (**B**) Dark-field image of (A) showing the overlapped regions and the outlines of each material (B). (**C**) The fabrication of Cr/Au contacts on Hall-bar geometry graphene and MoS_2 channels. (**D**) Optical image of the devices in (C) encapsulated by a large HfS_2 flake (black highlight). (**E**) Optical image of the corresponsing device after laser irradiation in the regions marked as HfO_x. The final device configurations include (i) hBN-graphene-HfO_x (ii) hBN-MoS_2-HfO_x (iii) SiO_2-MoS_2-HfO_x. (**F**) Resistance vs backgate voltage for graphene encapsulated between HfO_x and hBN for different sweep rates. (**G**) Conductance vs backgate voltage for hBN-MoS_2-HfO_x. (**H**) and SiO_2-MoS_2-HfO_x (H) measured with different sweep rates. (F to H) top: Device measurement schematics. Arrows indicate the gate voltage sweep direction.

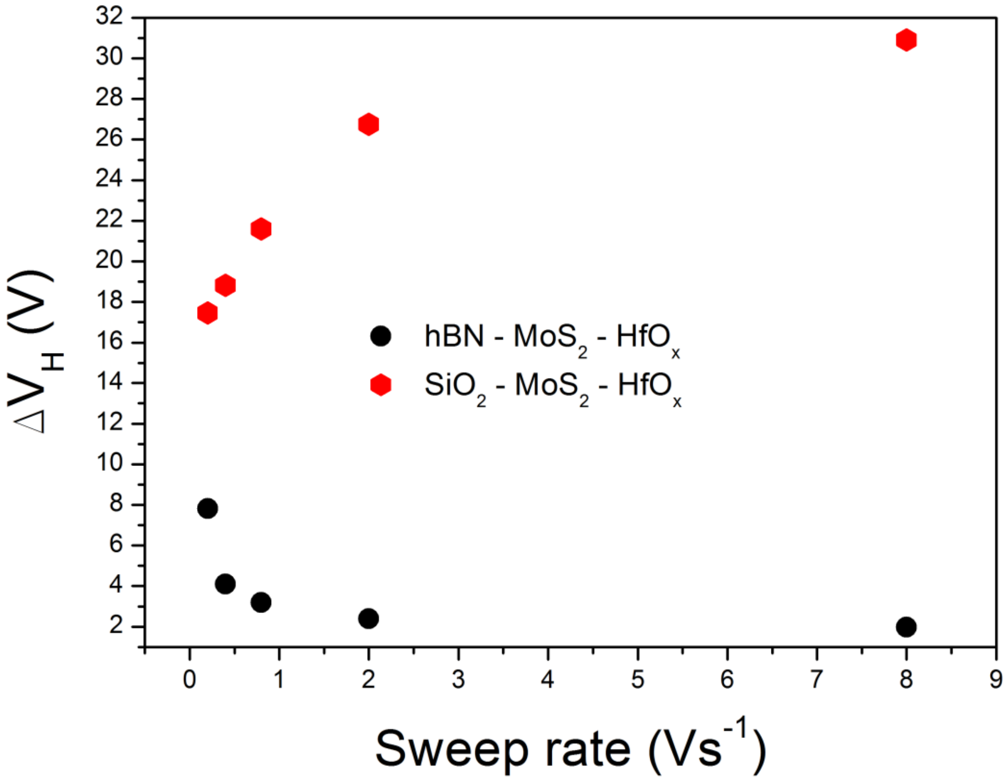

Fig. S7. Comparison of hysteresis width (ΔV_H) as a function of sweep rate for the hBN-MoS_2-HfO_x and SiO_2-MoS_2-HfO_x devices.

Section S6. Further examples of ReRAM elements with titanium adhesion layer

Figure S8 shows further examples of ReRAM heterostructure devices based on laser oxidised HfO_x barrier material. Figure S8 (A-C) shows resistive switching properties for a 20 nm thick HfO_x barrier at different compliance levels from 4 mA to 6 mA. While fig. S8D shows an example of a 5nm HfO_x barrier. Figure S8E shows an example of a 5nm resistive switching element which displayed a large $R_{SET}/R_{RESET} = 10^4$. In this device the RESET compliance level was pulsed to 1 mA to break the filament.

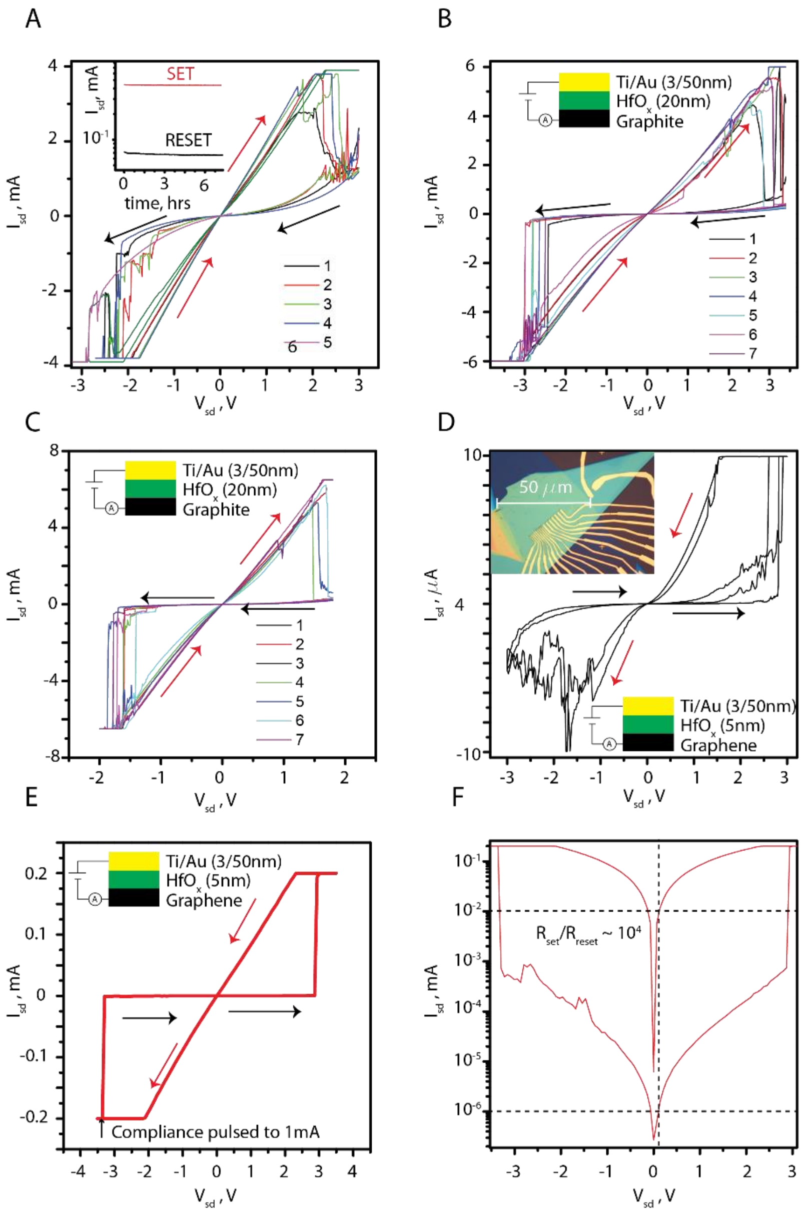

Fig. S8. Additional ReRAM devices. (**A** to **C**) Resistive switching data for several cycles for a heterostructure consisting of graphite-HfO_x-Ti/Au for increasing current compliance levels. (**D**) Example of resistive switching for a thinner 5nm HfO_x barrier. (**E** and **F**) Resistive switching device displaying large R_{SET}/R_{RESET}.

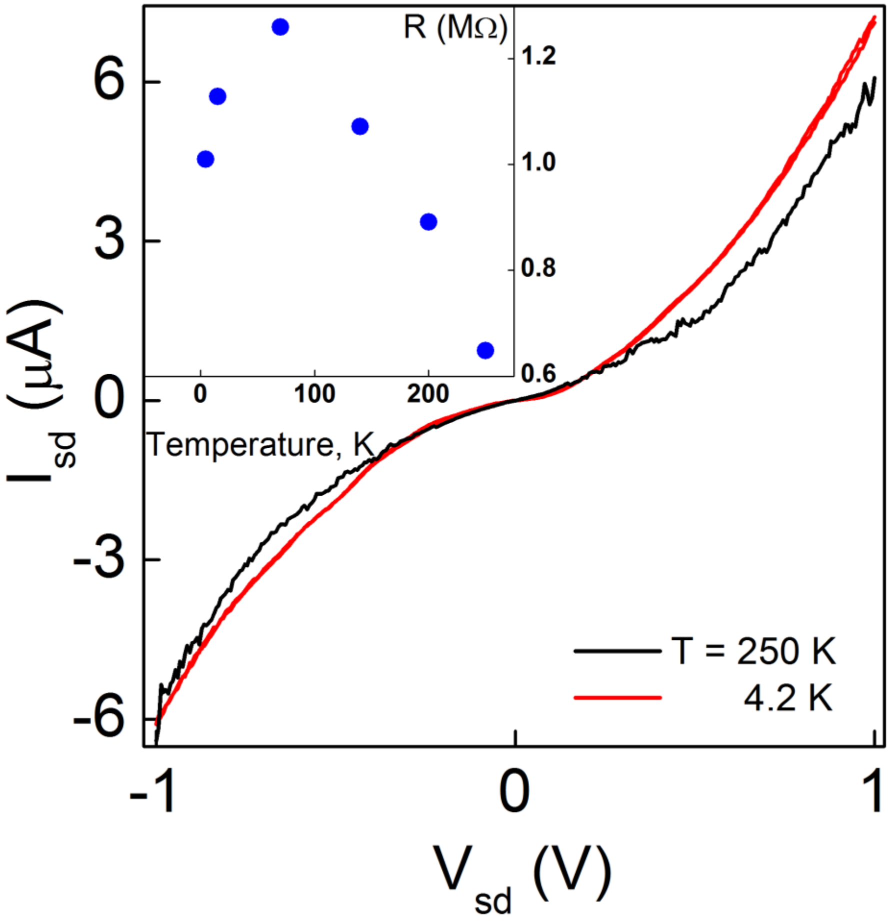

Fig. S9. Temperature dependence of the resistance for a graphite-HfO$_x$-Cr/Au vertical structure with *t* < 3 nm tunnel barriers. I_{sd}-V_{sd} characteristics measured at T = 4.2 and 300 K (Junction area: 8 mm^2). Inset: extracted low bias junction resistance vs temperature.

Section S7. Additional optoelectronic device data

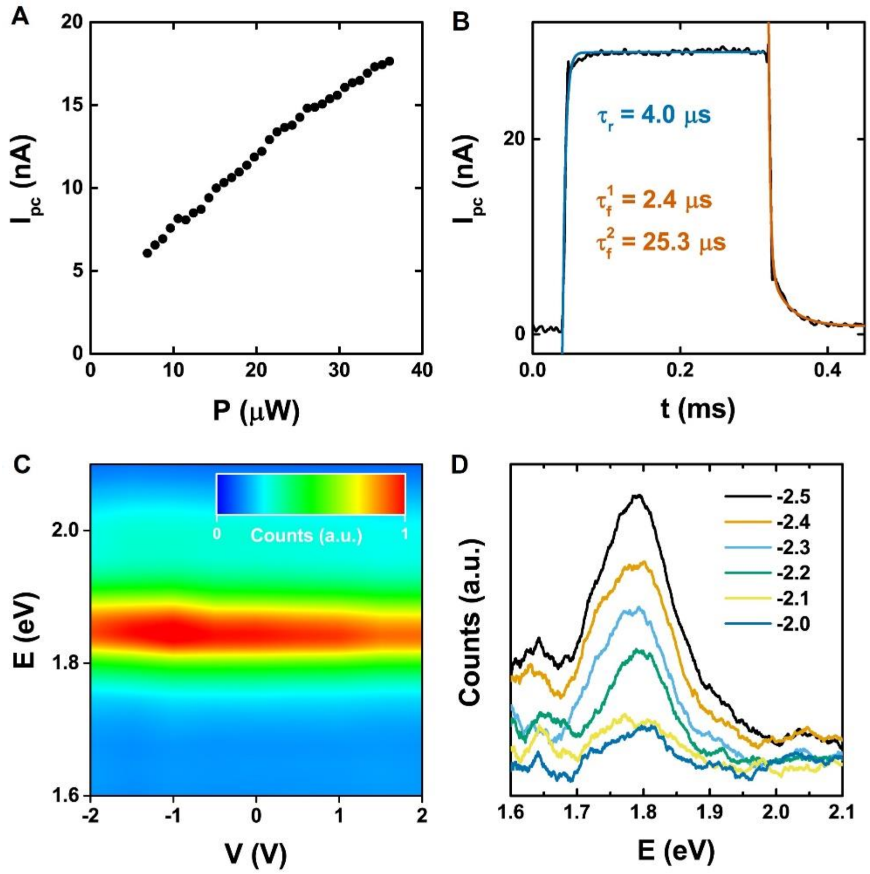

Fig. S10. Additional optoelectronic characterization. (**A**) Power dependence of the generated photo-current recorded at V_{sd} = -1 V. (**B**) Single oscillation of the photo-current. The rise (fall) times have been extracted by fitting the data, black line, with exponential decays with one (two) time constants. (**C**) PL intensity for different applied bias voltages. (**D**) EL spectra for different bias voltages extracted from the contour map shown in Fig. 6D of the main text.

In the Fig. 6, we identified the active area of the HfO_x QW through photocurrent mapping. A hotspot in the photocurrent is seen which corresponds to region in which the graphene electrodes overlap the HfO_x encapsulated MoS_2 flake. This localized photocurrent (Ipc), fig. S10A, has a non-linear power dependence which has been previously attributed to absorption saturation or electric field screening by the photoexcited carriers in MoS_2 (*61*). We observe an external quantum efficiency (EQE) of ($\eta = \frac{I_{pc}}{q}\frac{h\nu}{P} \sim 0.002\ \%$) smaller than previous works (*61*) which we anticipated due to the low absorption of monolayer MoS_2, the increased confinement of charges in the HfO_x QW, and off-resonance excitation. Furthermore, the low EQE demonstrates that there is no significant gain mechanism present in our device. This corroborates with the rise and fall time analysis, fig. S10B, which reveals an exponential decay with two-time constants, similar in magnitude. Therefore, it is unlikely that one results from long lived charge trapping – a common mechanism for photoconductive gain. As a result, this conclusion supports our claim of the formation of a clean oxide with few impurity states, crucial for the creation of a quantum well.

Aside from photocurrent generation, the extraction of carriers also manifests as a bias dependence of the photoluminescence (PL). For positive bias voltages the PL intensity decreases to a minimum at 2 V. As we increase the bias the Fermi level of the bottom graphene electrode aligns with the conduction band of MoS_2 favoring the extraction of photoexcited carriers preventing their recombination and quenching the PL. Similarly, as we sweep the bias to negative values we observe first a peak in PL intensity followed by a decrease with the peak located away from zero likely due to asymmetry in the thickness of the two barriers and the doping of top and bottom graphene.

Upon increasing the bias to more negative values ($V < -2$ V) we begin to observe electroluminescence (EL) as seen in Fig. 6E and discussed in the main text. In fig. S10D we present EL spectra taken at 0.1 V intervals showing the emergence of the main peak at 1.8 eV.
